# Supplementary material for: Novel Driver Strength Index highlights important cancer genes in TCGA PanCanAtlas patients
Source: PeerJ. 2022 Aug 11;10:e13860. doi: 10.7717/peerj.13860 (PMC9375969; doi:10.7717/peerj.13860)
Supplement: Supplemental Information 2 [file peerj-10-13860-s002.zip › COHORTS/genes plots/detailed/2021_8_16_14_9_distribution_events_detailed_males_2.pdf]

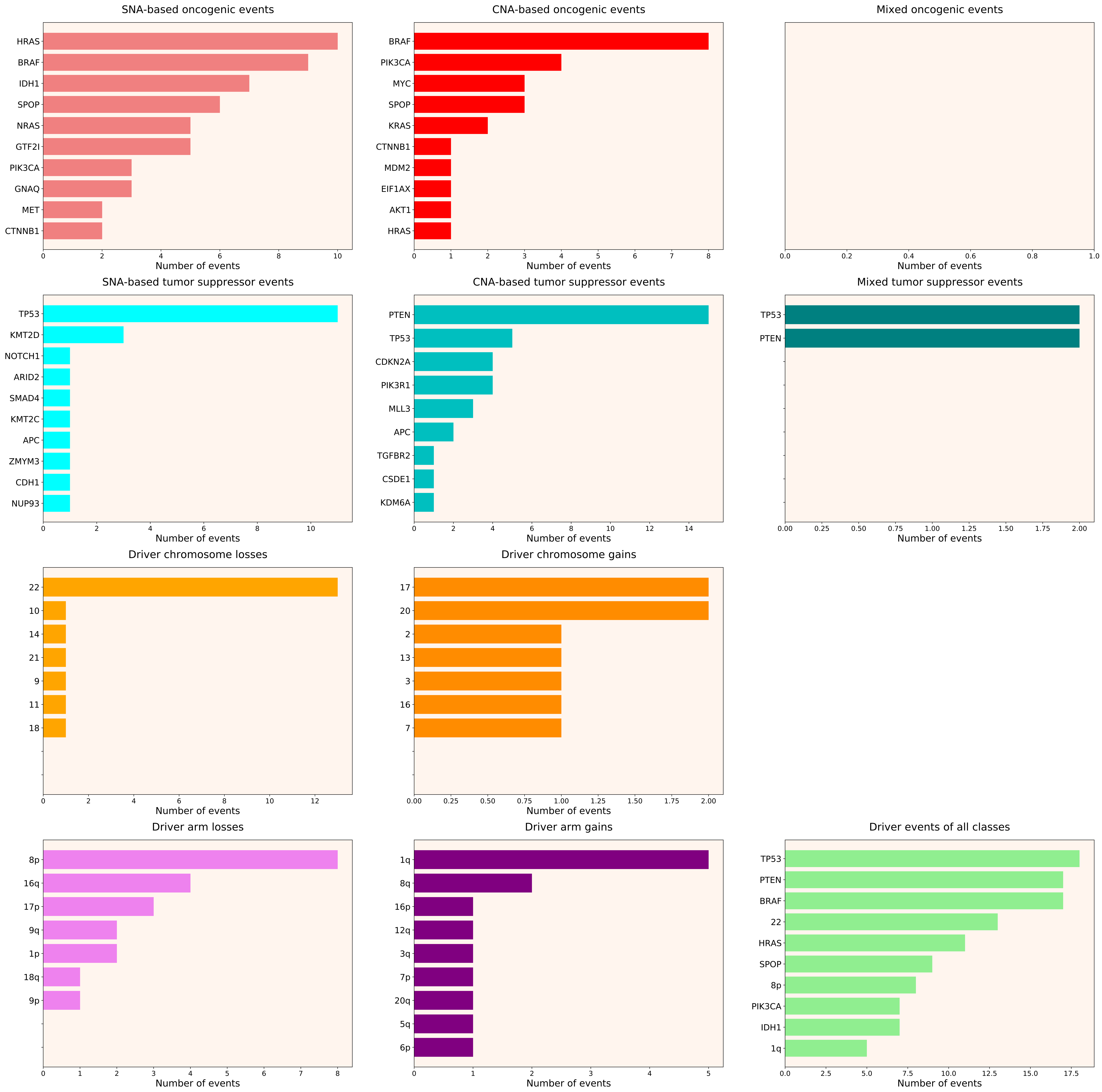
SNA-based oncogenic events
HRAS
BRAF
IDH1
SPOP
NRAS
GTF2I
PIK3CA
GNAQ
MET
CTNNB1
0 2 4 6 8 10
Number of events
CNA-based oncogenic events
BRAF
PIK3CA
MYC
SPOP
KRAS
CTNNB1
MDM2
EIF1AX
AKT1
HRAS
0 1 2 3 4 5 6 7 8
Number of events
Mixed oncogenic events
0.0 0.2 0.4 0.6 0.8 1.0
Number of events
SNA-based tumor suppressor events
TP53
KMT2D
NOTCH1
ARID2
SMAD4
KMT2C
APC
ZMYM3
CDH1
NUP93
0 2 4 6 8 10
Number of events
CNA-based tumor suppressor events
PTEN
TP53
CDKN2A
PIK3R1
MLL3
APC
TGFBR2
CSDE1
KDM6A
0 2 4 6 8 10 12 14
Number of events
Mixed tumor suppressor events
TP53
PTEN
0.00 0.25 0.50 0.75 1.00 1.25 1.50 1.75 2.00
Number of events
Driver chromosome losses
22
10
14
21
9
11
18
0 2 4 6 8 10 12
Number of events
Driver chromosome gains
17
20
2
13
3
16
7
0.00 0.25 0.50 0.75 1.00 1.25 1.50 1.75 2.00
Number of events
Driver arm losses
8p
16q
17p
9q
1p
18q
9p
0 1 2 3 4 5 6 7 8
Number of events
Driver arm gains
1q
8q
16p
12q
3q
7p
20q
5q
6p
0 1 2 3 4 5
Number of events
Driver events of all classes
TP53
PTEN
BRAF
22
HRAS
SPOP
8p
PIK3CA
IDH1
1q
0.0 2.5 5.0 7.5 10.0 12.5 15.0 17.5
Number of events
